# Influence of gender and education on cocaine users in an outpatient cohort in Spain

**DOI:** 10.1038/s41598-021-00472-7

**Published:** 2021-10-22

**Authors:** Nerea Requena-Ocaña, María Flores-Lopez, Alicia San Martín, Nuria García-Marchena, María Pedraz, Juan Jesús Ruiz, Antonia Serrano, Juan Suarez, Francisco Javier Pavón, Fernando Rodríguez de Fonseca, Pedro Araos

**Affiliations:** 1 Laboratorio de Medicina Regenerativa (LMR), Unidad de Gestión Clínica de Salud Mental, Instituto de Investigación Biomédica de Málaga (IBIMA), Hospital Regional Universitario de Málaga, Avda. Carlos Haya 82, sótano, 29010 Málaga, Spain; 2 Departamento de Psicobiología, Facultad de Psicología, Universidad Complutense de Madrid, Campus de Somosaguas, 28223 Pozuelo de Alarcón, Madrid, Spain; 3 Institut D, Investigació en Ciències de la Salut Germans Trias i Pujol (IGTP), Unidad de Adicciones-Servicio de Medicina Interna, Campus Can Ruti, Carrer del Canyet s/n, 08916 Badalona, Spain; 4 Centro Provincial de Drogodependencias (CPD) de Málaga, Diputación de Málaga, C/Ana Solo de Zaldívar, no 3, 29010 Málaga, Spain; 5 Department of Anatomy, Legal Medicine and History of Science, School of Medicine, University of Malaga, Boulevard Louis Pasteur 32, 29071 Málaga, Spain; 6 Instituto de Investigación Biomédica de Málaga (IBIMA), Unidad de Gestión Clínica del Corazón, Hospital Universitario Virgen de la Victoria de Málaga, Planta 5ª-Sección Central, Malaga, Spain; 7 Centro de Investigación Biomédica en Red Enfermedades Cardiovasculares (CIBERCV), Instituto de Salud Carlos III, Calle de Melchor Fernández Almagro, 3, 28029 Madrid, Spain; 8 Departamento de Psicobiología y Metdología de las CC del Comportamiento, Facultad de Psicología, Universidad de Málaga, Campus de Teatinos s/n, 29071 Málaga, Spain

**Keywords:** Psychology, Human behaviour

## Abstract

Gender significantly influences sociodemographic, medical, psychiatric and addiction variables in cocaine outpatients. Educational level may be a protective factor showing less severe addictive disorders, longer abstinence periods, and better cognitive performance. The aim was to estimate gender-based differences and the influence of educational level on the clinical variables associated with cocaine use disorder (CUD). A total of 300 cocaine-consuming patients undergoing treatments were recruited and assessed using the Psychiatric Research Interview for Substance and Mental Diseases according to the Diagnostic and Statistical Manual of Mental Disorders, Fourth Edition, Text Revision. Women developed CUD later but exhibited more consumption of anxiolytics, prevalence of anxiety disorders, eating disorders, and major depressive disorders. Alcohol and cannabis use disorders were more frequent in men. A predictive model was created and identified three psychiatric variables with good prognosis for distinguishing between women and men. Principal component analysis helped to describe the different profile types of men and women who had sought treatment. Low educational levels seemed to be a risk factor for the onset, development, and duration of CUD in both genders. Women and men exhibited different clinical characteristics that should be taken into account when designing therapeutic policies. The educational level plays a protective/risk role in the onset, development and progression of CUD, thus prolonging the years of compulsory education and implementing cognitive rehabilitation programmes could be useful.

## Introduction

Cocaine is a psychoactive substance whose consumption causes a strong health, social, and economic impact^1,2^. This substance is the most widely used illegal stimulant in Europe. In recent years, Spain has occupied the second place in the EU with respect to cocaine consumption^1^.

In Spain, 10.9% of adults (15–64 years) have used cocaine throughout their lives, 2.5% in the last year, and 1.1% in the last month^1,3^. Particularly, in Andalusia (Southern of Spain), the prevalence of admissions to treatment for cocaine use has increased from 4591 in 2014 to 5827 patients in 2018^4^.

Drug use has always been more prevalent in men than in women. This fact resulted from the male social and cultural role that allowed access much easier and the strong stigma around consumption in women^5,6^. At present, equal rights have allowed a rapid expansion of the consumption of different addictive substances among young women^6^, a reality that has already been reflected in the reports of school surveys.

Regarding to gender (considered as the sex [man or woman] reported by the patient when interviewed) and prevalence of psychoactive substance use in the last year, drug consumption is more widespread in men than in women except for hypnosedatives (62.4%) and opioid analgesics (56.6%)^3,7^. In the last years there was an increase of cocaine use for both genders, although is still more pronounced among men^7^. However, women who use cocaine suffer more social discrimination, have lower educational levels, higher unemployment rates, worse socioeconomic status, and worse health conditions. Instead, men exhibit greater prevalence of legal or criminal problems^8–10^.

In recent years, studies have addressed gender-based differences in patients with CUD^5,8^. Prevalence, use onset, progression of the disorder, relapses, and response to treatment have been found to vary between women and men^11,12^. Studies have indicated that women have lower rates of cocaine use^1,8,13^ and attend treatment centres to a lesser extent^1,14^. Furthermore, despite the fact that women initiate cocaine use later^5,15^ they are as likely as men to develop dependence after the first use^16^ and exhibit greater severity of addiction^8,14^. Women experience accelerated progression from the onset of consumption to the pathological use of cocaine^5,9^. This earlier process, called ‘telescoping’, has also been observed with other addictive substances such as alcohol, cannabis, and opiates^5^. In addition, women have higher relapse rates after stressful and/or depressive events^17^.

Taking into consideration the factors that contribute to worse CUD prognoses, it is worth mentioning that women exhibit greater predisposition to certain psychiatric comorbidities^5,10^. Specifically, women who use cocaine experience more anxiety, mood, and eating disorders^8,14,18^. On the other hand, antisocial personality disorders and alcohol and cannabis use disorders are more frequent among men who use cocaine, with earlier onset and more years of use than among women^19,20^.

Moreover, it is important to point out the occurrence of biases in the legal prescription of psychotropic medication to women, especially anxiolytics^21^. If we consider that such medication for primary psychiatric disorders loses effectiveness as drugs are used in combination, the management of these comorbid patients becomes much more difficult. For example, the efficacy of antidepressants is reduced by concomitant substance use^22^. Furthermore, benzodiazepines are prescribed over long periods of time, even though their prolonged use is not recommended due to the risk of dependence, which contributes to the appearance of disorders derived from the use of anxiolytics disguised by a legal route^21^.

In addition, some authors have indicated that the development of skills and experiences in patients with substance use disorders—such as academic achievements, occupational level, leisure activities, and social support—have been related to less serious addictive disorders, longer periods of abstinence, and better cognitive performances^23^. However, very few studies have explored the effects of these variables on cocaine-use onset and the development of CUD. One study indicated that cocaine-use patterns in European students were strongly related to socioeconomic status and educational levels, as well as school absenteeism and low level of leisure reading^24^. In addition, the most frequent negative consequences exhibited by these students who used substances of abuse were being absent from classes, memory problems, and low academic performance^25^. In another study conducted with an adolescent population, the authors observed that educational achievements had very important weight in the progression towards substance use disorders^26^. Furthermore, gender discrimination has been consistently associated with the use of illicit drugs and substance use disorders among women in the US, with those with an educational level below secondary occupying a position of greater risk^27^.

In light of the abovementioned factors, and given the need to promote differential therapeutic approaches between women and men, as well as prevention measures through educational level, the main objectives of the present study were to: (1) estimate the differences between women and men with respect to sociodemographic characteristics, other substances of abuse and psychiatric comorbidities; (2) determine the effect of gender and educational level on cocaine-related variables; (3) create a predictive model capable of discriminating between women and men, based on the aforementioned variables that can allow differential diagnoses; and (4) describe the different gender-based profiles in patients treated for cocaine use.

## Method

### Study design and cohort

This is a retrospective, descriptive and observational study conducted with a cohort of 300 cocaine users undergoing outpatient treatment centres divided into two groups according to gender (women *vs.* men). Abstinent patients were recruited in different drug addiction outpatient treatment centres (Málaga, Spain).

The inclusion criteria to be eligible for the present study were individuals aged over 18 years; cocaine users in the abstinence phase; being under outpatient treatment; and willingness to participate by signing an informed consent form. The chosen patients were evaluated to diagnose lifetime CUD and others psychiatric comorbidities based on DSM-IV-TR. Exclusion criteria included the presence of severe cognitive alterations and being in an acute psychotic episode in the active phase, which would not allow the normal development of the clinical assessment.

The present study fits within the framework of projects promoted by the Red de Trastornos Adictivos [RTA] (Addictive Disorders Network), an entity financed by the Instituto de Salud Carlos III (ISCIII), belonging to the Ministerio de Ciencia e Innovación of Spain. The ethical aspects of the core project (Proteomics of Cocaine Addiction: Central and Peripheral Biomarkers of Addiction) were approved by the Ethics and Clinical Research Committee of the Regional University Hospital of Malaga, respecting the ethical principles for medical research on human subjects adopted in the World Medical Association Declaration of Helsinki (64th WMA General Assembly, Fortaleza, Brazil, 2013). The assessment process was carried out by a team of clinical psychologists who had specialised and accredited training in psychiatric assessments.

### Measuring instruments

The Spanish version of the Psychiatric Research Interview for Substance and Mental Diseases (PRISM) was used to collect sociodemographic data and assess psychiatric disorders according to DSM-IV-TR criteria. PRISM is a semi-structured interview with good psychometric properties in the evaluation of substance use disorders and in the main comorbid psychiatric disorders related to the substance use population (Kappa coefficient between 0.66 and 1.00)^28^. First module of questions assesses the history of substance consumption, and second module assesses twenty Axis I and two Axis II disorders that are more prevalent in this population. One of the most important characteristics of this instrument is that it allows differentiating primary or independent mental disorders from substance-induced disorders^29,30^. The unidimensionality of the DSM-IV-TR criteria for CUD was used to determine cocaine trait severity combining the seven dependence criteria (for diagnosis of dependence three or more co-occurring symptoms in a 12-month period are required) and the four abuse criteria (one symptom is necessary for diagnosis of abuse).

### Procedure

A member of the therapeutic team collaborated in each outpatient treatment centre for recruitment of the participants. This person was in charge of informing about the existence of the study and inviting patients who had requested treatment at some point to participate. If they met the established inclusion criteria, the patients were referred to the team of clinical psychologists. In this way, the latter were in charge of summoning the patients and travelling through the different outpatient treatment centres in the Province of Malaga, Spain, where the clinical assessments were performed once the informed consent form had been signed by the participants.

The psychiatric assessments were performed in the same morning and could last between two and three hours. Finally, each interview was recorded in a database designed for the study. The interviews were conducted between 2010 and 2020**.**

### Study variables

The variables of the present study were: (a) Sociodemographic variables: age; marital status; number of children; educational level; occupational status; and cell/prison; (b) Medical, therapeutic and psychopharmacological treatment variables: chronic medical problems; psychiatric/psychological support; attendance at twelve-step groups; psychotropic medications (last 12 months); anxiolytics; antidepressants; antipsychotics; abstinence maintenance treatment; and disulfiram; (c) Psychiatric comorbidity variables: total psychiatric comorbidity; mood disorders; major depressive disorder; dysthymia; manic episode; hypomanic episode; cyclothymia; anxiety disorders; generalised anxiety disorder; obsessive compulsive disorder; post-traumatic stress disorder; seizure panic disorder; specific phobias; social phobia; psychotic disorders; schizophrenia; schizophreniform disorder; unspecified psychotic disorder; brief psychotic disorder; eating disorders; anorexia; bulimia; personality disorders; borderline personality disorder; antisocial personality disorder; attention-deficit hyperactivity disorder; and (d) Cocaine-related variables: severity criteria; age at onset of use; age at dependence development; length of abstinence; number of abstinences; duration of CUD; alcohol use disorder; cannabis use disorder; and sedatives use disorder.

### Statistical analysis

The results were analysed using the statistical programme SPSS version 19.0 (IBM SPSS Statistics for Windows. Armonk, NY: IBM Corp). All data in the tables are expressed as numbers and percentage of subjects [N (%)] or means and standard deviations (SD). First, the differences in the qualitative variables were determined using Fisher's exact test [Chi-square test (χ^2^)]. Despite the small number of women in the study, we analysed gender differences in sociodemographic, psychiatric comorbidity and cocaine-related variables using different chi-square test according to the number of categories and the sample size:

- Chi-square when there was more than two categories and a high sample size for each group [N > 5(%)].
- Chi-square by trend or linear by linear association when there was more than two categories and a small sample size for each group [N < 5 (%)].
- Fisher's exact test for two categories and a small sample size.

The normal distribution of the variables was assessed using Lilliefors corrected Kolmogorov–Smirnov test. As the continuous variables of the study did not meet the assumption of normality, statistical analyses were performed using non-parametric Mann–Whitney U test for comparisons between two groups, and Kruskal–Wallis test for comparisons of more than two groups. For *post-hoc* analyses we performed Dunn’s multiple comparison test.

To determine the variables that were able to discriminate between women and men, a binary logistic regression analysis was performed using Pearson's Chi-square (χ^2^) test, meeting the Hosmer–Lemeshow test. We assayed multicollinearity by examining Tolerance and Variance Inflation Factor (VIF). The cut off value for Tolerance was > 0.10 and < 10 for VIF. Sociodemographic variables, variables related to cocaine use patterns and psychiatric comorbidity variables were included in the equation. Finally, exploratory factor analysis with varimax rotation and bivariate relationships (correlation) was performed to determine the different profiles of patients who attended outpatient treatment for cocaine use, differentiated by gender. Only variables with a factorial load of at least 0.3 (sharing at least 10% of the variance with a factor) were used for interpretation. A *p* value less than 0.05 was considered statistically significant.

The discriminative power of the logistic model and the thresholds of certain variables were evaluated by Receiver Operating Characteristics (ROC) analysis considering the Area Under the Curve (AUC). A *p* value less than 0.05 was considered statistically significant.

## Results

### Sociodemographic characteristics in cocaine users cohort

The description of the sociodemographic variables of the sample composed of 300 patients is illustrated in Table 1. Among patients who attended outpatient treatment for cocaine use, the 84.7% were men and the mean age was 35.6 years (SD = 9.1); whereas the 15.3% were women, with a mean age of 37.5 years (SD = 8.2). We did not find statistically significant differences in sociodemographic variables when comparing women and men.

**Table 1:** Sociodemographic characteristics in total, men, and women cocaine users cohort.

Variable		Total N = 300	Men N = 254	Women N = 46	*p* Value
Age [media (SD)]	Years	35.89 (8.95)	35.60 (9.07)	37.52 (8.19)	0.072^a^
Marital status [N (%)]	Single Married/cohabiting Divorced/separated Widower	131 (43.70) 106 (35.30) 61 (20.30) 2 (0.70)	110 (43.30) 88 (34.60) 56 (22.00) –	21 (45.70) 18 (39.10) 5 (10.90) 2 (4.30)	0.702^b^
Children [mean (SD)]	Number	1.02 (1.26)	1 (1.26)	1.13 (1.22)	0.412^b^
Educational level [N (%)]	Primary/elementary Secondary University	72 (24.00) 195 (65.00) 33 (11.00)	60 (23.60) 167 (65.70) 27(10.60)	12 (26.10) 28 (60.90) 6 (12.00)	0.800^b^
Occupational status [N (%)]	Employed Medical sick leave Unemployed Retired Homework	105 (35.00) 21 (7.00) 160 (53.30) 11 (3.70) 3 (1.00)	89 (35.00) 20 (7.90) 134 (52.80) 10 (3.90) 1 (0.40)	16 (34.80) 1 (2.20) 26 (56.50) 1 (2.20) 2 (4.30)	0.450^b^
Prison/detained [N (%)]	Yes No	125 (41.70) 175 (58.30)	111 (43.7) 143 (56.3)	14 (30.4) 32 (69.6)	0.105^b^

*N* number of patients, *SD* standard deviation, *%* percentage, *BMI* Body Mass Index.

^a^*P*-value of the the Mann–Whitney U test.

^b^*P*-value of the Chi-Square test.

In addition, the 90.7% of the total sample were patients diagnosed with CUD while the 9.3% were patients who did not meet the diagnosis of CUD according to DSM-IV-TR.

### Medical, therapeutic, and psychopharmacological variables according to gender in cocaine users cohort

We performed an analysis to assess gender bias in prescribed psychotropic drugs. There were significant differences (χ^2^ = 11.63, *p* = 0.001) in the percentage of total consumption of psychotropic medication between women and men (84.1% vs. 56.9%). These differences were especially relevant for consumption of anxiolytics (χ^2^ = 8.53, *p* = 0.005), since women took them more compared to men (63.3% vs 39.9%) (Supplementary Table 1S).

### Comorbid psychiatric disorders in CUD patients according to gender

We investigated the influence of gender in the psychiatric comorbidity associated with CUD. There were significant differences in the prevalence of anxiety disorders (χ^2^ = 15.17, *p* < 0.001) and eating disorders (χ^2^ = 10.65, *p* = 0.007) between genders being more prevalent in women than in men (45.5% vs 18.5% and 11.4% vs 1.8%, respectively) (Supplementary Table 2S).

The variables related to specific comorbid psychiatric disorders are illustrated in Table 2. We observed significant gender differences for generalised anxiety disorder (χ^2^ = 7.88, *p* = 0.0013) and post-traumatic stress disorder (χ^2^ = 16.96, *p* < 0.001) being more prevalent in women than in men (22.7% vs 8.3% and 25% vs 5.7%, respectively). Similarly, we found significant differences in the diagnosis of anorexia (χ^2^ = 20.946, *p* = 0.001) and bulimia (χ^2^ = 3.74, *p* = 0.021) being more frequent in women compared to men (9.1% vs 1.8% and 6.8% vs 0%, respectively). On the other hand, there were significant gender differences in the presence of comorbid alcohol use disorder (χ^2^ = 8.77, *p* = 0.004) and cannabis use disorder (χ^2^ = 7.16 *p* = 0.022), being more prevalent in men than women (60.5% vs. 36.4% and 28.2 vs 11.4%, respectively).

**Table 2:** Prevalence of psychiatric comorbidity of total, men, and women with CUD (DSM-IV-TR).

Variable			Cocaine use disorder			
			Total N = 272	Men N = 228	Women N = 44	*p* Value
Mood disorders	Major depressive disorder [N (%)]	Yes No	73 (26.80) 198 (72.80)	61 (26.80) 166 (72.80)	12 (27.30) 32 (72.70)	< 0.999
	Dysthymia [N (%)]	Yes No	10 (3.70) 261 (96)	8 (3.50) 219 (96.10)	2 (4.50) 42 (95.50)	0.667
	Manic episode [N (%)]	Yes No	5 (1.80) 265 (97.40)	5 (2.20) 221 (96.90)	– 44 (100)	< 0.999
	Hypomanic episode [N (%)]	Yes No	4 (1.50) 268 (98.50)	3 (1.30) 225 (98.70)	1 (2.30) 225 (98.70)	0.508
	Cyclothymia [N (%)]	Yes No	4 (1.50) 266 (97.80)	2 (0.90) 224 (98.20)	2 (4.50) 42 (95.5)	0.126
Anxiety disorders	Generalized anxiety disorder [N (%)]	Yes No	29 (10.70) 241 (88.60)	19 (8.30) 207 (90.80)	10 (22.70) 34 (77.30)	**0.013**
	Obsessive compulsive disorder [N (%)]	Yes No	3 (1.10) 267 (98.20)	2 (0.90) 224 (98.20)	1 (2.30) 43 (97.70)	0.415
	Post traumatic stress disorder [N (%)]	Yes No	24 (8.90) 247 (90.80)	13 (5.70) 214 (93.90)	11 (25.00) 33 (75.00)	** < 0.001**
	Panic attack [N (%)]	Yes No	11 (4) 260 (95.60)	8 (3.50) 219 (96.10)	3 (6.80) 41 (93.20)	0.257
	Specific phobias [N (%)]	Yes No	8 (2.90) 263 (96.70)	5 82.20) 222 (97.40)	3 (6.80) 41 (93.20)	0.124
	Social phobias [N (%)]	Yes No	1 (0.40) 271 (99.60)	1 (0.40) 227 (99.60)	– 44 (100.00)	< 0.999
Psychotic disorders	Schizophrenia [N (%)]	Yes No	– 272 (100)	– 228 (100)	– 44 (100)	–
	Schizophreniform disorder [N (%)]	Yes No	3 (1.10) 269 (98.90)	2 (0.90) 226 (99.10)	1 (2.30) 43 (97.70)	0.412
	Delusional disorder [N (%)]	Yes No	4 (1.50) 268 (98.5)	3 (1.30) 225 (98.70)	1 (2.30) 43 (97.70)	0.508
	Schizoaffective disorder [N (%)]	Yes No	3 (1.10) 269 (98.90)	2 (0.90) 226 (99.10)	1 (2.30) 43 (97.70)	0.412
	Unspecified psychotic disorder [N (%)]	Yes No	5 (1.80) 267 /98.20)	4 (1.80) 224 (98.2)	1 (2.30) 43 (97.70)	0.589
	Brief Psychotic Disorder [N (%)]	Yes No	14 (5.10) 258 (94.90)	13 (5.70) 215 (94.30)	1 (2.30) 43 (97.70)	0.703
Eating disorders	Anorexia [N (%)]	Yes No	4 (1.50) 267 (98.20)	– 228 (100)	4 (9.10) 40 (90.90)	**0.001**
	Bulimia [N (%)]	Yes No	7 (2.60) 264 (97.10)	4 (1.80) 223 (97.80)	3 (6.80) 41 (93.20)	**0.021**
Personality disorders	Antisocial personality disorder [N (%)]	Yes No	60 (22.10) 210 (77. 20)	53 (23.50) 173 (76.50)	7 (15.90) 37 (84.10)	0.325
	Borderline personality disorder [N (%)]	Yes No	52 (19.10) 218 (80.10)	41 (18) 185 (81.10)	11 (25) 33 (75)	0.300
Other substance use disorders	Alcohol use disorder [N (%)]	Yes No	154 (56.60) 118 (43.40)	138 (60.50) 90 (39.50)	16 (36.40) 28 (63.60)	**0.004**
	Cannabis use disorder [N (%)]	Yes No	68 (25.10) 203 (74.90)	64 (28.20) 163 (71.80)	5 (11.40) 39 (88.60)	**0.022**
	Sedative use disorder [N (%)]	Yes No	28 (10.30) 244 (89.70)	24 (10.50) 204 (89.50)	4 (9.10) 40 (90.90)	0.999

*N* number of patients, *%* percentage.

*P*-value of the Chi-Square test. Significant *P*-value in bold.

Regarding the type of substance use disorder, we found statistically significant gender differences according to the type of disorder (primary, induced or both) with respect to major depressive disorder (χ^2^ = 3.91, *p* = 0.048). Specifically, women had more major depressive disorders of both types (induced and primary) than men (33.30 vs 2.30%, *p* = 0.012) (Supplementary Table 3S).

### Impact of gender and educational level on cocaine-related variables in CUD patients

We analysed the influence of gender and educational level in the variables associated with cocaine use. As shown in Table 3, we observed the effect of gender for the variable ‘age at development of dependence’ (U = 3463.50, *p* = 0.010, η2 = 0.025). Women developed cocaine dependence 2.5 years later than men, at the age of 28.1 years for women and 25.6 years for men.

**Table 3:** Differences in cocaine consumption patterns in patients with CUD according to gender.

Variable		Cocaine use disorder N = 272		
		Men N = 228	Women N = 44	*p* Value
Severity criteria [mean (95% CI)]	Criteria (0–11)	8.09 (7.68–8.49)	7.94 (7.10–8.79)	0.174
Age at onset of use [mean (SD)]	Years	20.13 (6.99)	20.45 (7.12)	0.454
Age at dependence development [mean (SD)]	Years	25.55 (7.49)	28.14 (6.31)	**0.010**
Length of abstinence [mean (SD)]	Days	192.34 (661.03)	147.36 (216.20)	0.488
Number of abstinences [mean (SD)]	Number	1.51 (0.96)	1.34 (0.94)	0.220
Duration of CUD [mean (SD)]	Years	7.72 (6.84)	7.46 (5.81)	< 0.999
Telescoping effect [mean (SD)]	Years	5.66 (5.48)	6.19 (6.23)	0.656

*%* percentage, *CI* confidence interval, *SD* standard deviation, *CUD* cocaine use disorder.

*P* value for Mann–Whitney U test. Significant *P*-value in bold.

As shown in Table 4, we found an effect of educational level for the variables ‘age at the onset of use’ (H = 8.23, *p* = 0.016), ‘age at development of dependence’ (H = 14.20, *p* = 0.001) and ‘duration of CUD’ (H = 7.84, *p* = 0.020). CUD patients with primary education started cocaine use 6.22 years earlier than patients with university studies (U = 172, *p* = 0.006, η2 = 0.124). Similarly, CUD patients with primary education developed cocaine dependence 6.52 years earlier than those with university education (U = 445, *p* < 0.001, η2 = 0.134) and patients with secondary educational had cocaine dependence 4.64 years earlier than those with university education (U = 1334, *p* = 0.003, η2 = 0.046). Patients with primary education presented 3.47 years longer of CUD than those with secondary (U = 4079.50, *p* = 0.008, η2 = 0.031).

**Table 4:** Differences in cocaine consumption patterns in patients with CUD according to educational level.

Variable		Cocaine use disorder N = 272			
		Primary N = 69	Secondary N = 178	University N = 25	*p* Value
Severity criteria [mean (95% CI)]	Criteria (0–11)	8.03 (7.32–8.74)	8.17 (7.70–8.63)	7.85 (6.73–8.97)	0.473
Age at onset of use [mean (SD)]	Years	18.33 (5.76)	20.35 (7.04)	24.93 (8.28)	**0.016**
Age at dependence development [mean (SD)]	Years	24.12 (6.88)	26.01 (7.20)	30.64 (7.88)	**0.001**
Length of abstinence [mean (SD)]	Days	199.42 (454.97)	173.56 (681.67)	219.75 (316.75)	0.127
Number of abstinences [mean (SD)]	Number	1.40 (0.89)	1.44 (0.933)	2.00 (1.25)	0.074
Duration of CUD [mean (SD)]	Years	10.28 (9.04)	6.81 (5.34)	6.52 (5.52)	**0.020**
Telescoping effect [mean (SD)]	Years	5.06 (6.16)	5.88 (5.18)	6.71 (7.52)	0.157

*%* percentage, *CI* confidence interval, *SD* standard deviation, *CUD* cocaine use disorder.

*P* value for the Education effect (Kruskal–Wallis H). Significant *P*-value in bold.

Additionally, we explored multiple paired comparisons between gender and education for cocaine use patterns. Regarding the woman group, we found significant differences in ‘age at the onset of use’ (H = 8.81, *p* = 0.012). Women with primary education started cocaine use 7.5 years earlier than those with secondary education (U = 24, *p* = 0.007, η2 = 0.256). Among men group, we observed significant differences in ‘age at the onset of use’ (H = 11.22, *p* = 0.004) and ‘age at development of dependence’ (H = 14.95, *p* = 0.001). Thus, men with primary education started cocaine use three years earlier than those with secondary education (U = 1752.50, *p* = 0.005, η2 = 0.049), and 6.6 years earlier than those who had attended university (U = 106, *p* = 0.007, η2 = 0.142). Similarly, men with primary education had cocaine dependence three years earlier than those with secondary educational (U = 3089.50, *p* = 0.008, η2 = 0.036) and 7 years earlier than those with university education (U = 281.50, *p* = 0.001, η2 = 0.148). Moreover, men with secondary education had cocaine dependence 4.33 years earlier than those with university education (U = 941.50, *p* = 0.013, η2 = 0.038). Among those with primary education, men started cocaine use 7.7 years earlier than women (U = 31.50, *p* = 0.001, η2 = 0.230) and develop cocaine dependence 6.25 years earlier (U = 135.50, *p* = 0.003, η2 = 0.132).

Telescoping effect was measured by subtracting the age at onset of cocaine use and the age at development of CUD. However, we did not find significant gender or educational differences.

### Gender-based prediction variables in CUD patients

We generated a binary logistic regression model to evaluate the potential of sociodemographic, psychiatric and cocaine related variables as exploratory variables to discriminate between male and female patients. The variables included in the first step were those in which were found gender differences: “educational level” (primary, secondary, university), “psychotropic medication”, “anxiolytics”, “alcohol use disorder”, “cannabis use disorder”, “anxiety disorders”, “posttraumatic stress disorder”, “generalised anxiety disorder”, “eating disorders”, “bulimia”, “type of major depressive disorder” (primary, induced or both), “age at onset of use”, and “age at dependence development”. Anorexia was removed from the analysis for not having a representative sample for men. All variables met the statistical assumptions of multicollinearity.

**Figure 1:**
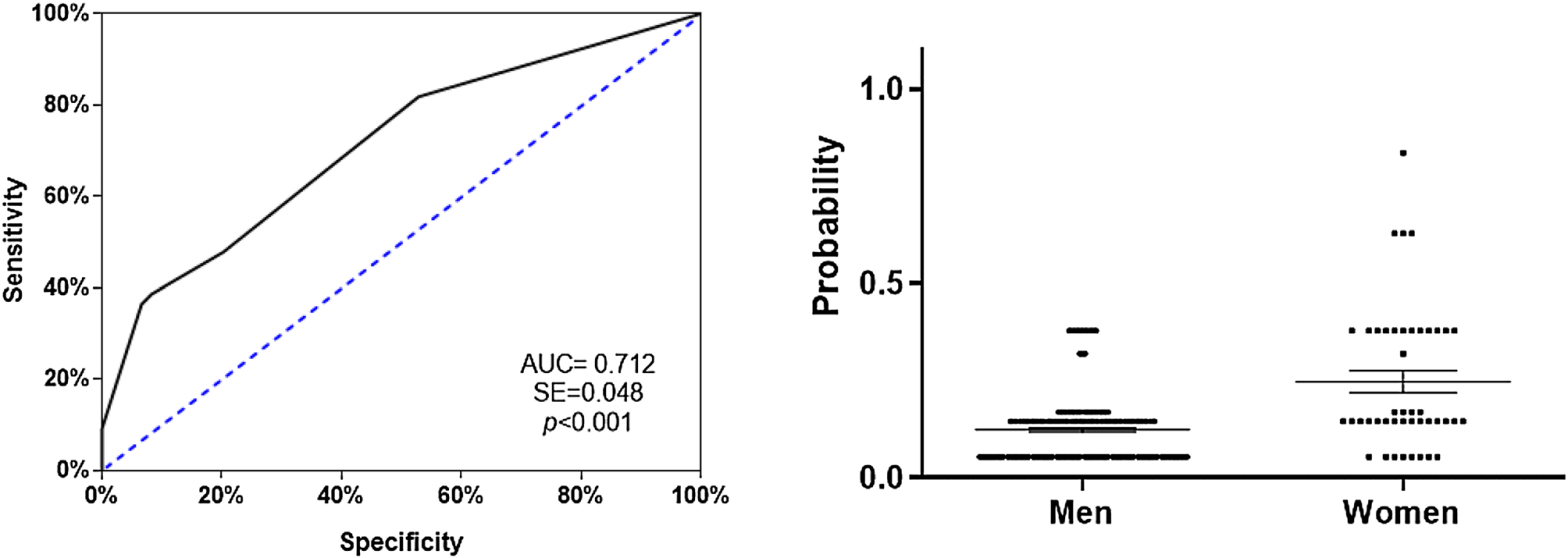
Analysis of the ROC curve (left) using the forward stepwise (conditional) to assess potential of sociodemographic variables and patterns of cocaine use as exploratory variables [area under the curve (AUC = 0.712 (0.048) with *P* < 0.001] to discriminate between men and women patients (N = 204). Dispersion points (right) for the predictive model of logistic regression between men and women. The scatter plot of the predictive probabilities for the patients with CUD indicated that the means were significantly different between both groups (U = 2870. *p* < 0.001).

Model was prepared using the forward stepwise method and the predictive covariates were restricted to three, which were “alcohol use disorder”, “anxiety disorders”, and “eating disorders” (Supplementary Table 4S). Hosmer–Lemeshow test indicated good calibration (χ^2^ = 1.579, *p* = 0.454) and was able to explain the variation of the dependent variable in 17.3% of the cases according to the Nagelkerke R2 method. It had a classification percentage of 86.8%, showing a high sensitivity for classifying men (100%) and women (12.9%) CUD patients. As seen in Fig. 1, the ROC curve analysis indicated an AUC = 0.712, which represented medium discrimination power. The scatter plot of the predictive probabilities for the patients with CUD indicated that the means were significantly different between both groups (U = 2870, *p* < 0.001).

### Complex gender-based profile in women attending outpatient treatment for cocaine use

**Figure 2:**
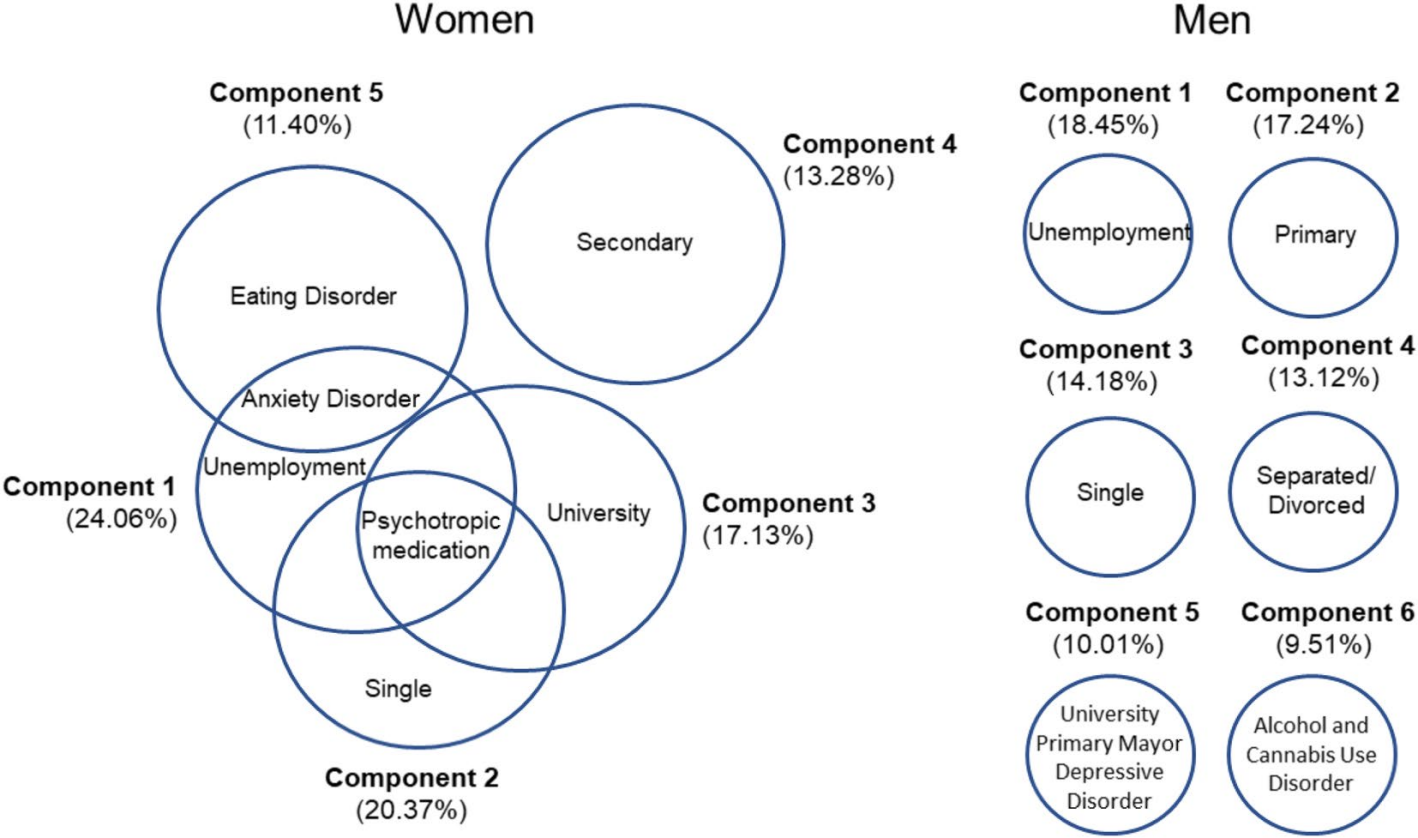
Exploratory principal component analysis for women and men. Differential gender profiles in patients attending outpatient treatment for cocaine use (N = 300). Six components together explained 82.7% of the variance that allowed describing the different profiles of men who attended outpatient treatment for cocaine use (Figure 2, right). Five components together explained 84.5% of the variance that allowed describing the different profiles of women who attended outpatient treatment for cocaine use (Figure 2, left).

We performed a principal component analysis to evaluate the potential of sociodemographic, psychiatric and cocaine related variables as descriptive variables to explain the different gender profiles of women and men who attend to clinical cocaine treatments. Six components together explained 82.7% of the variance that allowed describing the different profiles of men who attended outpatient treatment for cocaine use (Fig. 2, right). Component 1 explained 18.4% of the total variance and was associated with unemployment (0.923). Component 2 explained 17.2% of the total variance and it was associated with primary education level and had high factor load (0.907). Component 3 explained 14.2% of the total variance and it was related to the single status, with a high factor load (0.857). Component 4 explained 13.1% and was closely associated with divorced, with a high factorial load (0.994). Component 5 explained 10% of the total variance and was related to university studies and primary major depressive disorder, with a high factor load (0.610 and 0.844, respectively). Finally, component 6 explained 9.5%, and was associated with alcohol use disorder and cannabis use disorder, achieving high factor loads (0.761 and 0.728, respectively).

Five components together explained 84.5% of the variance that allowed describing the different profiles of women who attended outpatient treatment for cocaine use (Fig. 2, left). Component 1 explained 24.1% of the total variance. It was associated with unemployment, psychotropic medication, and anxiety disorders, with high factor loads (0.908, 0.637, and 0.322, respectively). Component 2 explained 20.4% of the total variance and was associated with the single status and consumption of psychotropic medication (0.908 and 0.377, respectively). Component 3 explained 17.1% of the total variance and it was associated with the level of secondary education and showed a high factor load (0.883). Component 4 explained 13.3% of the total variance, being associated with university studies and psychotropic medication, with a high factor load (0.900 and 0.401, respectively). Component 5 explained 11.4% of the total variance and was closely associated with anxiety disorders and eating disorders, exhibiting a high factor load (0.720 and 0.813, respectively).

## Discussion

This study adds to the growing literature on gender differences in CUD and incorporate information on sociodemographic, medical, psychopathological and cocaine consumption variables. Moreover, it adds a new point of view that investigates how educational achievements can affect the onset of use, the development of dependence and the duration of cocaine addiction in a different way among men and women. In addition, this study shows a comprehensive analysis of gender profiles based on models from patients attending treatment for cocaine use.

There is high prevalence of psychiatric comorbidities associated with CUD, which may coexist with other substance use disorders or other psychiatric disorders^31–34^. Regarding its association with gender-based differences, in our study the most prevalent disorders in women were anxiety disorders, with generalised anxiety disorder and post-traumatic stress disorder being highly common, as also described in other findings^15,20,32^. In the women population, traumatic experiences such as having suffered child abuse and sexual abuse are factors that have been considered predictive for the development of CUD^32,35^. Thus, these women are more likely to have post-traumatic stress disorder, and more likely to have any anxiety disorders in comparison to men^9^. On the other hand, we observed that the women also showed high prevalence of eating disorders and exhibited more depressive disorders of both, induced and primary nature, throughout their lives in comparison to men, as has also been described in other studies^8,14,20^.

Concerning men population, we found a frequency of antisocial personality disorders that were similar to by other authors^15,20^. Similarly, we observed that the coexistence of another substance use disorders throughout life was 64.3%, with alcohol use disorders and cannabis use disorders being more common, as reported by other authors^14,33^. Men are more likely to develop polydrug use of most substances except anxiolytics^3^. There is a biological and social basis for understanding the comorbidity between alcohol use disorder and CUD, since pathological alcohol use enhances cocaine use and vice versa^36^. It is worth noting that the processes of gender violence are more evident in individuals who have concomitant pathological use of these two substances^37^.

Thus, if the pathological use of other substances of abuse (especially alcohol), as well as evident lack of effective pharmacological therapies are added to those psychiatric comorbidities, the evolution of these patients consequently worsens, being more vulnerable to relapse and distancing them from primary health resources and deepening their social stigmatisation^38,39^.

Related to the variables associated with cocaine consumption, we observed that women developed cocaine dependence two years later than men (28 vs. 26 years), as other authors have also indicated^5,15^. However, we did not find a faster process in women from the first use of cocaine to the development of CUD (telescoping effect), nor did we observe greater severity of addiction in comparison to men, as described in other studies^40,41^. In addition, we did not find gender-based differences in other variables such as the age at onset of use, duration of abstinence, number of abstinences, or the duration of the disorders, as reported by other authors^9,15^.

Despite the several gender-based differences related to drug use have been described, healthcare services do not consider the need for a different medical approach for women and men, in spite of demonstrating deserving characteristics of special attention^18^. Our model showed that women and men had different clinical characteristics that should be reflected when designing therapeutic policies. Therefore, it is important to take into account the results of the logistic regression analysis carried out in which appear as predictors the variables: “alcohol use disorder”, “anxiety disorders”, and “eating disorders”. We believe that this class of models—based on sociodemographic variables, patterns of cocaine use, and associated psychiatric comorbidities—could provide information about the aetiology and the progression of the addiction, individually and specifically. They could be useful guides for professionals both at preventive and clinical levels. In this way, preventive measures could be determined and better therapeutic strategies will be developed to improve the quality of treatment.

Furthermore, our results indicated that the profiles of women who sought treatment were different and more complex. The most repeated male profile was that of unemployed men, whereas the most frequent female profile was that of unemployed women who consumed psychotropic medication and had anxiety disorders. This fact means that they had come to the centres in worse psychiatric state than men. In addition, they were at risk of social exclusion and vulnerability. In other words, a triple stigma was observed: being a woman, addiction, and mental disorder.

This characteristic profile in women could indicate a delay in entering the treatment circuits, and we consider that this could be motivated by social, cultural, and medical reasons. Women consume in a less exposed way, since addictions continue to be poorly accepted socially, thus constituting a reason for stigma and implying less family and social support in the female^6,42^. Requests for treatment decreases due to the legal or social repercussions that the consumption of substances could cause to women during the periods of motherhood and education of their children. It is also important to emphasise the pressure exerted by the family members so that the treatments are as brief as possible and the woman can return to perform the household chores, often sacrificing treatment^43^. These aspects explain the low rate of women’s admissions to treatments, since they only represented 15% of the total sample of our study, like other studies^20,44^.

In addition, the three most frequent profiles of women seeking treatment for cocaine use had the use of psychotropic medication as a common factor, even though two of the profiles did not exhibit any correlation with a specific psychiatric symptom, which could reflect the medical tendency to prescribe this type of psychotropic drugs to women in great proportion^21^.

Finally, there are very few studies that relate educational levels to the use of cocaine and much less with its interaction with gender. We observed the protective effect that the educational level exerts on the development of cocaine addiction: the onset and development of CUD occurred earlier when patients had a lower educational level and these had a longer duration of CUD, whereas patients with university studies started use and developed CUD later. This has also been observed in alcohol, since young people who had dropped out of secondary education or university studies were at higher risk of developing abuse in adult life, compared to those who had completed secondary school or university^45^.

The CUD has also been related to executive dysfunctions that compromise efforts to initiate or maintain abstinence and affects the functionality and therapeutic effectiveness of patients^46,47^. Regarding the relationship between gender and educational level, women and men with low educational level started cocaine use much earlier than those with secondary studies. This is particularly important to women with primary education, since they could have a higher risk of gender discrimination and illicit drug use^27^. However, the age at onset of cocaine use and the development of CUD is still earlier in men compared to women who have a low educational attainment.

All this evidence places the educational level as a risk factor or a protective factor depending on the academic achievements, occupying a fundamental role in the appearance and course of addictive disorders. Low educational level can predispose to the emergence of earlier and more severe CUD. While high educational level can prevent or delay the onset of consumption and the subsequent development of CUD, as well as provide better cognitive skills that can promote therapeutic success. Therefore, prolonging the years of compulsory education, as well as implementing cognitive rehabilitation programmes in treatment centres could be extremely useful both for the prevention and for the evolution of patients^48^.

In conclusion, women and men have different clinical characteristics that should be considered when designing therapeutic policies for patients with CUD. The educational level plays a fundamental role in the onset and progression of CUD, which is why it is essential to focus attention on it. Therefore, the progressive equality between women and men in the consumption of addictive substances leads us to be even more rigorous in the development of gender-based therapies.

## Conclusion remarks

- Women have lower percentages of attendance at treatment centres.
- Women consume more prescribed psychotropic medication, especially benzodiazepines.
- Women have higher prevalence of anxiety disorders and eating disorders, particularly posttraumatic stress disorder, generalised anxiety disorder, anorexia, and bulimia.
- Men have higher rates of other substance use disorder as alcohol and cannabis use disorders.
- Women develop cocaine dependence later than men (28 vs 26 years).
- Patients with primary and secondary educational level start use and develop cocaine dependence early than those with university studies.
- Patients with primary studies suffer more years of CUD than those with secondary educational level.
- Women with primary educational attainment start cocaine use much earlier than those with secondary studies.
- Men with primary educational level start use and develop cocaine dependence early than those with secondary and university studies. Similarly, men with secondary education develop cocaine dependence early than those with university studies.
- Among patients with primary educational level, men start use and develop cocaine dependence much earlier than women.
- Alcohol use disorder, anxiety disorders and eating disorders demonstrated to be good variables to classify between men and women (86.8%).
- The profiles of women who sought treatment for cocaine use were more complex than those of men. They come to the centres in worse psychiatric and social states.

## Limitations and prospects

The present study has several limitations that should be considered in further research. First, the small number of female populations was relevant from a clinical perspective, though relatively small from a statistical perspective. Second, these results can only be interpreted in a clinical setting and not in the general population; therefore, caution should be exercised when extrapolating the data to other population settings. Third, we can mention the recall bias of the interviewed patients, due to the use of retrospective diagnostic tools.

In the future, we should consider a series of prospects for improving this type of studies addressing populations with CUD. Firstly, longitudinal studies could be conducted in order to assess the evolution and prognosis of psychiatric comorbidities and severity of the addictions. Also, these studies could assess the cognitive abilities before and after treatments, particularly according to the educational levels of the patients. Secondly, the number of female patients should be increased in the studies. Gender-based comparisons would be much more robust from a statistical point of view. Finally, visibility of this under-diagnosis reality in the population with CUD could be increased with the aim of designing specific therapies according to the characteristics of each gender.

## Supplementary Information

Supplementary Information.
