# Supplementary material for: Influence of gender and education on cocaine users in an outpatient cohort in Spain
Source: Sci Rep. 2021 Oct 22;11:20928. doi: 10.1038/s41598-021-00472-7 (PMC8536710; doi:10.1038/s41598-021-00472-7)
Supplement: Supplementary file 1 — Supplementary Information. [file 41598_2021_472_MOESM1_ESM.docx]

**SUPPLEMENTARY MATERIAL**

| **Supplementary Table 1S.** Medical, therapeutic, and psychopharmacological variables according to gender in cocaine users cohort | | | | | |
| --- | --- | --- | --- | --- | --- |
| **VARIABLE** | | **TOTAL N=300** | **MEN N=254** | **WOMEN N=46** | ***p* Value** |
| **Chronic medical problem**  **[N (%)]** | *Yes*  *No* | 67 (24.40)  210 (75.80) | 52 (22.20)  182 (77.80) | 15 (34.90)  28 (65.10) | 0.178 |
| Psychiatric / psychological support  **[N (%)]** | *No*  *Outpatient*  *Hospitalized*  *Both* | 133 (45.70)  124 (42.60)  6 (2.10)  28 (9.60) | 117 (47.40)  104 (42.10)  4 (1.60)  22 (8.90) | 16 (36.40)  20 (45.50)  2 (4.50)  6 (13.60) | 0.111 |
| **12-Step treatment**  **[N (%)]** | *Yes*  *No* | 88 (29.50)  210 (70.50) | 80 (51.50)  174 (68.50) | 8 (18.20)  36 (81.80) | 0.077 |
| **All psychotropic medication**  **[N (%)] (last 12 months)** | *yes*  *No* | 181 (60.90)  116 (39.10) | 144 (56.90)  109 (43.10) | 37 (84.10)  7 (15.90) | **0.001** |
| **Antidepressants**  **[N (%)] (last 12 months)** | *Yes*  *No* | 94 (32.80)  193 (67.20) | 76 (31.30)  167 (68.70) | 18 (40.90)  26 (59.10) | 0.225 |
| **Anxiolitics**  **[N (%)] (last 12 months)** | *Yes*  *No* | 125 (43.60)  162 (56.40) | 97 (39.90)  146 (60.10) | 28 (63.30)  16 (36.40) | **0.007** |
| **Antipsychotics**  **[N (%)] (last 12 months)** | *Yes*  *No* | 34 (11.90)  251 (88.10) | 26 (10.70)  216 (89.30) | 8 (18.60)  35 (81.40) | 0.197 |
| **Abstinence Maintenance Treatment ***  **[N (%)] (last 12 months)** | *Yes*  *No* | 38 (13.30)  247 (86.70) | 29 (12.00)  213 (88.00) | 9 (20.90)  34 (79.10) | 0.114 |
| **Disulfiram**  **[N (%)] (last 12 months)** | *Yes*  *No* | 33 (11.60)  252 (88.40) | 28 (11.60)  214 (88.40) | 5 (11.60)  38 (88.40) | 0.999 |
| **Abbreviations**: N: Number of patients. %: Percentage.  *P*-value of the Chi-Square test * Acamprosate, nalmefene, naltrexone | | | | | |

Supplementary Table 2S. Prevalence of total, men, and women comorbid psychiatric disorders in CUD patients according to gender (DSM-IV-TR)

| VARIABLE | | COCAINE USE DISORDER | | | |
| --- | --- | --- | --- | --- | --- |
|  |  | TOTAL N=272 | MEN N=228 | WOMEN N=44 | *p V*alue |
| Total psychiatric comorbidity  [N (%)] | *Yes*  *No* | 158 (58.30)  113 (41.70) | 130 (57.30)  97 (42.70) | 28 (63.60)  16 (36.40) | 0.726 |
| Mood disorder  [N (%)] | *Yes*  *No* | 79 (29.20)  192 (70.80) | 65 (28.60)  162 (71.40) | 14 (31.80)  30 (68.20) | 0.718 |
| Anxiety disorder  **[N (%)]** | *Yes*  *No* | 62 (22.90)  209 (77.10) | 42 (18.50)  185 (81.50) | 20 (45.50)  24 (54.50) | <0.001 |
| Psychotic disorder  [N (%)] | *Yes*  *No* | 32 (11.80)  239 (88.20) | 27 (11.90)  200 (88.10) | 5 (11.40)  39 (88.60) | <0.009 |
| Eating disorder  [N (%)] | *Yes*  *No* | 9 (3.30)  263 (96.70) | 4 (1.80)  224 (98.20) | 5 (11.40)  39 (88.60) | 0.005 |
| Personality disorder  [N (%)] | *Yes*  *No* | 86 (31.90)  184 (68.10) | 71 (31.40)  155 (68.60) | 15 (34.20)  29 (65.90) | 0.726 |
| ADHD in childhood  [N (%)] | *Yes*  *No* | 23 (8.50)  249 (91.50) | 20 (8.80)  208 (91.20) | 3 (6.80)  41 (93.20) | <0.999 |
| Other substance use disorder  [N (%)] | *Yes*  *No* | 175 (64.30)  97 (35.70) | 150 (65.80)  78 (34.2%) | 25 (56.80)  19 (43.20) | 0.303 |
| Abbreviations: N: Number of patients. %: Percentage. ADHD: attention deficit hyperactivity disorder  *P*-value of the Chi-Square test | | | | | |

Supplementaty Table 3S. Type of psychiatric comorbidity in CUD patients (DSM-IV-TR).

| VARIABLE | | **COCAINE USE DISORDER** | | | |
| --- | --- | --- | --- | --- | --- |
|  |  | **TOTAL N=272** | **MEN N=228** | **WOMEN N=44** | **p Value** |
| Mayor Depressive Disorder | *Primary*  *Induced*  *Both* | 22 (30.60)  44 (61.10)  6 (8.30) | 19 (31.30)  39 (65.00)  2 (3.30) | 3 (25.00)  5 (41.70)  4 (33.30) | 0.003 |
| Dysthymia | *Primary*  *Induced*  *Both* | 2 (22.20)  6 (66.70)  1 (11.10) | 2(28.60)  5 (71.40)  - | -  1 (50.00)  1 (50.00) | 0.124 |
| Hipomaniac Episode | *Primary*  *Induced*  *Both* | 1 (25.00)  2 (50.00)  1 (25.00) | -  2 (66.70)  1 (33.30) | 1 (100.00)  -  - | 0.135 |
| Cyclotymia | *Primary*  *Induced*  *Both* | -  3 (75.00)  1 (25.00) | -  2 (100.00)  - | 1 (50.00)  1 (50.00)  - | 0.248 |
| Generalised Anxiety Disorder | *Primary*  *Induced*  *Both* | 11 (37.90)  14 (48.30)  4 (13.80) | 8 (42.10)  8 (42.10)  3 (15.80) | 3 (30.00)  6 (60.00)  1 (10.00) | 0.655 |
| Panic Attack | *Primary*  *Induced*  *Both* | 3 (30.00)  7 (70.00)  - | 2 (28.60)  5 (71.40)  - | 1 (33.30)  2 (66.70)  - | 0.880 |
| Schizoaffective Disorder | *Primary*  *Induced*  *Both* | 1 (33.30)  2 (66.70)  - | 1 (50.00)  1 (50.00)  - | -  1 (100.00)  - | 0.386 |
| Schizophreniform Disorder | *Primary*  *Induced*  *Both* | 2 (40.00)  2 (40.00)  5 (20.00) | 2 (50.00)  1 (25.00)  1 (25.00) | -  1 (100.00)  - | 0.392 |
| Unspecified Psychotic Disorder | *Primary*  *Induced*  *Both* | 1 (20.00)  4 (80.00)  - | 1 (25.00)  3 (75.00)  - | 1 (20.00)  4 (80.00)  - | 0.576 |
| Abbreviations: N: Number of patients. %: Percentage.  *P*-value of the Chi-Square test | | | | | |

**Supplementary Table 4S.** Binary logistic regression model based on sociodemographic variables and patterns of cocaine use for gender-based in CUD patients

| VARIABLE | **COCAINE USE DISORDER**  **N=204** | | | | | |
| --- | --- | --- | --- | --- | --- | --- |
|  | **B** | **SE** | **W** | **Df** | ***p* Value** | **OR [95%CI]** |
| **Alcohol Use Disorder** | 1.098 | 0.445 | 5.827 | 1 | **0.016** | 3 [1.229 -7.318] |
| **Anxiety Disorders** | -1.282 | 0.426 | 9.055 | 1 | **0.003** | 0.278 [0.120-0.640] |
| **Eating Disorders** | -2.117 | 0.840 | 6.350 | 1 | **0.012** | 0.120 [0.023-0.635] |
| **Abbreviations**: B=coefficient. SE= Standard error. W= Wald’s test. df= degree freedom. OR= Odds Ratio. CI= Confidence Interval.  Variables which enter in step 1: Educational Level (primary, secondary, university), Psychotropic Medication, Anxiolytics, Alcohol Use Disorder, Cannabis Use Disorder, Anxiety Disorders, Posttraumatic Stress Disorder, Generalised Anxiety Disorder, Eating Disorders, Bulimia, type of Mayor Depressive Disorder (primary, induced, both), Age at onset of use, Age at dependence development. | | | | | | |
